# Length of Stay for Mental and Behavioural Disorders Postpartum in Primiparous Mothers: A Cohort Study

**DOI:** 10.3390/ijerph110403540

**Published:** 2014-03-27

**Authors:** Fenglian Xu, Marie-Paule Austin, Nicole Reilly, Lisa Hilder, Elizabeth A Sullivan

**Affiliations:** 1 National Drug and Alcohol Research Centre, University of New South Wales, Randwick, NSW 2031, Australia; 2 Perinatal and Women’s Mental Health Unit, St. John of God Health Care and School of Psychiatry, University of New South Wales, Burwood, NSW 2134, Australia; E-Mails: m.austin@unsw.edu.au (M.P.A.); n.reilly@unsw.edu.au (N.R.); 3 The Black Dog Institute, Hospital Road, Prince of Wales Hospital, Randwick, NSW 2031, Australia; 4 National Perinatal Epidemiology and Statistics Unit, School of Women’s and Children’s Health, University of New South Wales, Randwick, NSW 2031, Australia; E-Mails: l.hilder@unsw.edu.au (L.H.); e.sullivan@unsw.edu.au (E.A.S.)

**Keywords:** mental health, length of hospital stay, postpartum, data linkage

## Abstract

*Background*: Previous research showed that there was a significant increase in psychiatric hospital admission of postpartum mothers. The aim of the current study is to describe the length of hospital stays and patient days for mental and behavioural disorders (MBD) of new mothers in the first year after birth. *Method*: This was a cohort study based on linked population data between the New South Wales (NSW) Midwives Data Collection (MDC) and the NSW Admitted Patients Data Collection (APDC). The study population included primiparous mothers aged from 18 to 44 who gave birth between 1 July 2000 and 31 December 2005. The Kaplan–Meier method was used to describe the length of hospital stay for MBD. *Results*: For principal diagnoses of MBD, the entire length of hospital stay in the first year postpartum was 11.38 days (95% CI: 10.70–12.06) for mean and 6 days (95% CI: 5.87–6.13) for median. The length of hospital stay per admission was 8.47 days (95% CI: 8.03–8.90) for mean and 5 days (95% CI: 4.90–5.10) for median. There were 5,129 patient days of hospital stay per year for principal diagnoses of postpartum MBD in new mothers between 1 July 2000 and 31 December 2005 in NSW, Australia. *Conclusions*: MBD, especially unipolar depressions, adjustment disorders, acute psychotic episodes, and schizophrenia, or schizophrenia-like disorders during the first year after birth, placed a significant burden on hospital services due to long hospital stays and large number of admissions.

## 1. Introduction

Mental health morbidity in the perinatal period is a major public health issue with postpartum depression affecting up to 15 % of women [1,2]. A systematic review showed that as many as 6.5% mothers have a new episode of major depression during the first three months after delivery [3]. A cohort study in Danish population showed that the rate of first-time hospital admission for mental disorders in the first year postpartum was 0.1% including 0.6% in the first month after birth [4]. 

There were on average 85,648 women giving birth in NSW and 254,522 in Australia each year between 2000 and 2005 [5]. In Australia, there were 20 public psychiatric hospitals, 122 public acute hospitals with a psychiatric ward or unit, 26 private psychiatric hospitals and 234 government-operated community and residential mental health facilities in 2004–2005 [6]. During this period, there were 199,353 hospital separations for mental illness. Of these, public acute hospitals accounted for 73.8% of these separations, private hospitals accounted for 19.2% and public psychiatric hospitals accounted for 7.0%. For admitted patients, more than half of the mental health-related separations (58.6%) included specialised psychiatric care [6]. 

Australian hospital statistics between 2000 and 2006 showed that there were large numbers of patient days for mental and behavioural disorders (MBD) [7,8,9,10,11,12]. The average length of stay (ALOS) for overall hospital separations ranged between 3.3 and 3.7 days in Australia and from 3.6 and 4.0 days in New South Wales (NSW) between 2000 and 2005 [7,8,9,10,11,12]. The ALOS for MBD as principal diagnoses ranged from 11.0 to 13.0 days per separation in public hospitals and from 4.9 to 6.2 days per separation in private hospitals in Australia in the same time period [7,8,9,10,11,12]. Public hospitals included public psychiatric hospitals where the ALOS was much longer than private hospitals and public acute hospitals [6].

Postpartum women are a special population. Some literatures based on population data reported an increased trend of hospital admissions for MBD in the first year after birth [2,4,13]. The morbidity and costs associated with maternal MBD in the period is substantial for a mother and her family [14]. One small UK study (N = 206) conducted between 1997 and 1999 which examined the economic costs of health care for postnatal depression found a mean cost increase of 392 pounds per woman for overall postnatal care in women with (versus without) postnatal depression over the first 18 months post-partum [14]. However, there is little literature reporting the ALOS and patient days in the postnatal period [15]. 

To address the gap, we used population-based linked data to answer four specific questions: (1) What is the entire length of hospital stay in NSW for primiparous mothers in the first year postpartum? (2) What is the ALOS for MBD including those of specific diagnosed groups? (3) Which diagnoses contribute more to hospital admission and stays? (4) Is there any difference in the hospital admission and stay between principal and non-principal diagnoses of MBD? 

## 2. Methods

### 2.1. Study Population

The study subjects included all primiparous women aged 18–44 years who gave birth (including live and still births) in NSW between 1 July 2000 and 31 December 2005 and were admitted to hospital (including public and private hospitals) with a diagnosis of MBD in the first year after birth. 

### 2.2. Study Design

This is a population-based longitudinal study using linked data between the NSW Midwives Data Collection (MDC) and the NSW Admitted Patients Data Collection (APDC). MDC birth records from 1 July 2000 to 31 December 2005 were linked with APDC records between 1 July 2000 and 31 December 2006, so that hospital admissions for mothers could be followed up at least one year after birth. 

The MDC is a data collection for all live births and stillbirths of at least 20 weeks gestation or at least 400 grams birthweight in NSW. It covers all births in public and private hospitals as well as home births, and includes information on maternal characteristics, pregnancy, labour, delivery and neonatal outcomes. The APDC is a routinely collected census of all hospital separations and includes information on patient demographics, diagnoses and clinical procedures. It includes all patient hospitalisations in NSW public and private hospitals including psychiatric hospitals and day procedures. Since 1999, diagnoses for admissions have been coded according to the 10th revision of the International Statistical Classification of Diseases and Related Health Problems, Australian Modification (ICD-10-AM) [16]. 

Data linkage was performed by the NSW Department of Health Centre for Health Record Linkage (CHeReL) using probabilistic record linkage methods and choiceMaker software [17]. Identifying information from MDC and APDC datasets was included in the Master Linkage Key constructed by the CHeReL. At the completion of the process, each record was assigned a number, Person Project Number, to allow records for the same individual to be linked. Based on the 1,000 randomly selected sample of records, the false positive rate of the linkage was 0.3% and false negative < 0.5%. 

### 2.3. Definitions

Postpartum is defined as the period starting from the day of birth and ending 365 days later. Length of hospital stay is defined as the time span in days from hospital admission to separation of each hospitalisation in the first year postpartum. The lengths of hospital stay for mothers who were admitted and separated on the same day were counted as one day; those who separated in the next day were counted as two days, and so on.

Entire or accumulated length of hospital stay refers to the total length in days of hospital stay for an individual in the first year postpartum.

Principal diagnosis was the diagnosis which was chiefly responsible for the hospital admission [18]. 

Non-principal diagnosis is a condition or a complaint either coexisting with the principal diagnosis or arising during the hospitalisation. It includes stay and other diagnoses.

Stay diagnosis is an additional diagnosis and refers to the diagnosis that most influenced the length of stay in hospital [19]. 

### 2.4. Diagnosis of Mental and Behavioral Disorders

The diagnoses for each admission in this study have been coded according to the Australian modification to the World Health Organization ICD-10 Classification of Diseases and Related Health Problems (ICD-10-AM) [16]. Mothers with mental disorders were identified using ICD-10-AM diagnosis codes: (1) schizophrenia, schizophrenia-like disorder including schizoaffective disorders (ICD-10-AM: F20, 21, 22, 24, 25, 28, 29); (2) unipolar depressions (ICD-10-AM: F32 exclude 32.3, F53.0, F34.1, F38 exclude 38.0); (3) acute psychotic episodes: reactive, brief, affective (ICD-10-AM: F23, 32.3, 33.3, 39, 53.1); (4) bipolar affective disorders (ICD-10-AM: F30.0, 30.2, 30.8, 30.9, 34.0, 38.0, F31 exclude 31.7); (5) adjustment disorders (ICD-10-AM: F43); (6) anxiety disorders (ICD-10-AM: F40, 41,42); (7) personality disorders (ICD-10-AM: F60–69); (8) mental and behavior disorder due to substance use (ICD-10-AM: F10–19); (9) Remaining diagnoses (ICD-10-AM: F00–99 exclude the diagnoses above); (10) overall diagnosis of mental and behavioural disorders including psychiatric disorder and substance use disorder(ICD-10-AM: F00–99 ). 

### 2.5. Statistical Analysis

For the analysis of total length of hospital stay in the first year after birth, the duration of each hospital stay during the period was calculated firstly by using maternal age in days at discharge minus maternal age in days at admission. Then the duration of hospital stays in the first year postpartum were added together by using the function of aggregate data. 

Descriptive statistics were used to analyze the length of hospital stay and proportion of hospital admission. 

The duration between hospital admission and the separation for MBD were used for Kaplan–Meier analysis which describes mean and median of the length of hospital stay and 95% confidence interval (CI) for MBD. The time is from hospital admission to separation or the 365th day after birth. The event is separation. Log rank test were used for comparison. The analyses were conducted using IBM Statistical Package for Social Science Statistics 20 [20]. 

### 2.6. Ethics Approval

This study was approved by the NSW Population & Health Services Research Ethics Committee and the Human Research Ethics Committee of the University of New South Wales, Australia.

## 3. Results

From 1 July 2000 to 31 December 2005, there were 5,861 primiparous mothers aged 18–44 years who admitted to a NSW hospital (7,394 admissions) with diagnoses of MBD in the first year postpartum. Among them, 3,332 admissions were for principal diagnoses and 4,062 admissions for stay or other diagnoses.

The median number of days was a better estimate than the mean for central tendency because the distributions of the length of hospital stay in this study were skewed. However, the mean could reflect the impact of extremely long hospital stays and is commonly used in the literature [18,21,22]. As a result we included both means and medians in the results for comparison.

Table 1 shows the length of hospital stay of mothers with diagnoses of MBD. For principal diagnoses, the entire length of hospital stay in the first year postpartum was 11.38 days (95% CI: 10.70–12.06) for mean and 6 days for median (95% CI: 5.87–6.13). 

**Table 1:** Length of hospital stay of primiparous mothers with diagnosis or comorbidity of mental and behavioural disorders in the first year postpartum.

Measurement	Diagnosis	N ^a^	Patient Days	Mean ALOS			Median ALOS		
				Days	95% CI		Days	95% CI	
Entire length in the first year after birth	Principal	2,479	28,212	11.38	10.70	12.06	6	5.87	6.13
	Non-principal	3,679^b^	23,286	6.33	6.14	6.52	6	5.92	6.08
	Total	5,861	51,498	8.79	8.45	9.12	6	5.93	6.07
Length per admission	Principal	3,332	28,212	8.47	8.03	8.90	5	4.90	5.10
	Non-principal	4,062	23,286	5.73	5.57	5.89	5	4.92	5.08
	Overall	7,394	51,498	6.96	6.75	7.18	5	4.93	5.07

Notes: ^a^ Refers to number of mothers for entire length of hospital stay and to number of hospital admissions for length per admission. ^b^ Some mothers had more than one admission during postpartum period and the admissions may be with different diagnoses including principal or non-principal diagnoses. As a result there is some overlap in the number with principal and non-principal diagnoses.

The length of hospital stay per admission was 8.47 days (95% CI: 8.03–8.90) for mean and 5 days (95% CI: 4.90–5.10) for median. For mothers with diagnoses of MBD as stay or other diagnoses, the means of entire length of hospital stay (mean = 6.33 days, 95% CI: 6.14–6.52) and the length of hospital stay per admission (mean = 5.73 days, 95%CI: 5.57–5.89) were significantly shorter than those with principal diagnoses (*p* < 0.05). The medians for entire length of hospital stay and the length of hospital stay per admission were not significantly different with those of principal diagnoses (*p* > 0.05). However, stay or other diagnoses accounted for 55% of overall hospital admissions and 45% of patient days with MBD diagnoses. There were 9,363 (51,498/5.5) person days of hospital stay each year for MBD in new mothers in NSW including 5,129 person days for principal diagnoses and 4,234 person days for stay or other diagnoses. The denominator of 5.5 refers to the total follow up person years (5 years for mothers who gave birth between 2001 and 2005, 0.5 year for the mothers who gave birth between July and December of 2000).

**Figure 1:**
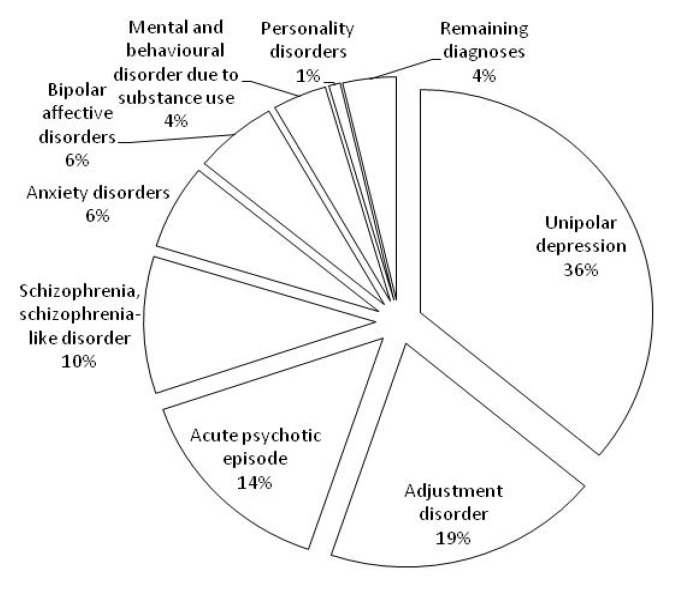
Percentage of patient days for principal diagnoses of mental and behavioural disorders 2000–2005, NSW.

Table 2 shows the hospital admissions and stays attributed to specific principal diagnoses in the first year postpartum between 1 July 2000 and 31 December 2005. Unipolar depressions accounted for the largest proportion of hospital admissions (37.55%) and patient days (36.03%) in the first year postpartum. Adjustment disorders accounted for the second largest proportion of hospital admissions (27.16%) and patient days (19.18%). Schizophrenia, schizophrenia-like disorders had the longest mean ALOS but not the longest median ALOS (22.40 days for mean, 95% CI: 15.91–28.89; 9.00 days for median, 95% CI: 4.30–13.70). Acute psychotic episodes accounted for the second longest hospital stay (18.73 days for mean, 95% CI: 16.06–21.40; 12 days for median, 95% CI: 9.31–14.69). The diagnoses above, including unipolar depressions, adjusted disorders, schizophrenia, schizophrenia-like disorders and acute psychotic episodes, contributed to four-fifth patient days and three-fourth hospital admissions for principal diagnoses of MBD. Figure 1 shows the proportions of patient days in details.

**Figure 2:**
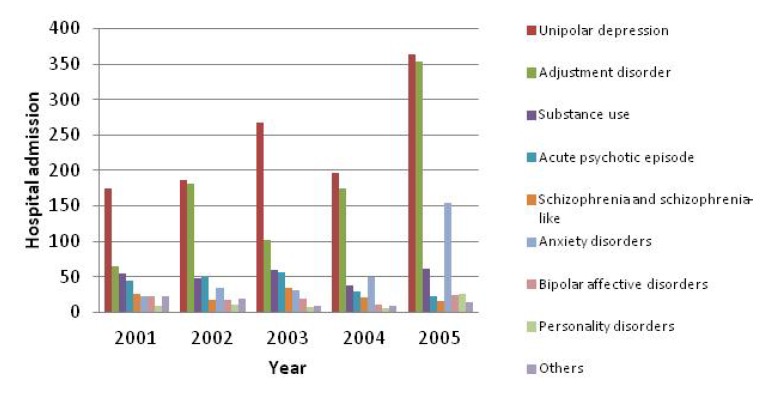
Hospital admissions for principal diagnoses of mental and behavioural disorders 2000–2005, NSW.

Table 3 shows an increase of patient days in 2005. It was due to the significant rise in hospital admissions for unipolar depressions, adjustment disorders and anxiety disorders (see Figure 2). Figure 2 does not include the hospital admissions in 2000 which covered only half-year admissions from 1 July to 31 December.

**Table 2:** Length of hospital stay of primiparous mothers with principal diagnoses of mental and behavioural disorders in the first year postpartum 2000–2005, NSW.

	Admissions		Sum		Mean ALOS			Median ALOS		
Diagnosis	n	%	(Patient days)	%	Days	95% CI		Days	95% CI	
Unipolar depression	1,251	37.55	10,166	36.03	8.13	7.55	8.71	5	4.83	5.17
Adjustment disorder	905	27.16	5,410	19.18	5.98	5.78	6.17	6	5.87	6.13
Acute psychotic episode	220	6.60	4,121	14.61	18.73	16.06	21.40	12	9.31	14.69
Schizophrenia, schizophrenia-like disorder	125	3.75	2,800	9.92	22.40	15.91	28.89	9	4.30	13.70
Anxiety disorders	303	9.09	1,736	6.15	5.73	5.08	6.38	5	4.84	5.16
Bipolar affective disorders	105	3.15	1,642	5.82	15.64	12.79	18.49	11	7.87	14.14
Mental and behavior disorder due to substance use	273	8.19	1,062	3.76	3.89	3.20	4.59	1	-	-
Personality disorders	61	1.83	225	0.80	3.69	2.11	5.27	1	-	-
Remaining diagnoses	89	2.67	1,050	3.72	11.80	7.83	15.76	5	3.15	6.85
Overall	3,332	100.00	28,212	100.00	8.47	8.03	8.90	5	4.90	5.10

**Table 3:** Length of hospital stay for principal diagnoses of mental and behavioural disorders in the first year postpartum, NSW.

		Sum	Mean ALOS			Median ALOS		
Year	Admissions	(Patient day)	Days	95% CI		Days	95% CI	
2000 ^a^	169	1,940	11.48	9.38	13.57	6	4.79	7.21
2001	441	4,353	9.87	8.46	11.28	5	4.56	5.44
2002	565	5,064	8.96	7.94	9.99	6	5.75	6.25
2003	587	4,788	8.16	6.77	9.55	5	4.75	5.25
2004	532	4,371	8.22	7.32	9.11	5	4.88	5.12
2005	1,038	7,696	7.41	6.83	7.99	6	5.85	6.15
Overall	3,332	28,212	8.47	8.03	8.90	5	4.90	5.10

Note: ^a^ The data in 2000 covered only half-year admissions from 1 July to 31 December.

## 4. Discussion

The latest National Survey of Mental Health and Wellbeing conducted in 2007 by the Australian Bureau of Statistics estimated that 3.2 million Australians, or 20% of the adult population, experienced symptoms of a mental disorder [23]. Expenditure on mental health services has been increasing on average by 6% per year (adjusted for inflation) since 2003 in Australia [23]. A survey in Victoria and South Australia in 2007 showed that at six months postpartum the proportion of women scoring above the “normal” range on the Depression Anxiety Stress Scales −21 was 12.7% for anxiety, 17.4% for depression and 8.1% for comorbid depression and anxiety [24]. Mental illness in the postnatal period significantly impacts the health of the whole family, including the mother, partner and child [1,25].

In NSW, separations for principal diagnoses of MBD (F00–99) accounted for 4.23–4.40% of total hospital admissions between 2000 and 2005 [7,8,9,10,11,12]. The studies showed that the separations for MBD principal diagnoses in the first year postpartum accounted for 0.51% of all separations with MBD as the principal diagnoses in NSW in 2001; 0.66% in 2001 and 2003; 0.58% in 2004; and 1.08% in 2005 [7,8,9,10,11,12]. The number of postpartum separations increased significantly in 2005 compared with previous years (*p* < 0.05). This rise cannot be explained by a single factor. There was a 5% increase in the NSW birth rate in 2005 [26]. The effect of changes to Australian Coding Standards for ICD10AM is more difficult to quantify. Standards released in 2003 extended the diagnosis of postpartum depression (F53) from six weeks to one year after birth and provided a fifth character expansion for depressive disorders (F32) to code conditions in the postpartum period [27]. A limited extract of ICD10AM codes used in residential mothercraft centres that advocated the use of a non-specific principal diagnosis (Z76.8) was discontinued after 2004 [28].

Length of hospital stay was associated with facility type and diagnoses [21]. A study based on population data between 1996 and 2001 in the United States (US) showed that patients treated in mental health inpatient facilities had a significantly longer ALOS (10.5 days) than patients in general inpatient facilities (3.5 days) [21]. A study on substance use treatment in the US showed that the median number of days in treatment for women with substance use in pregnancy and postpartum was eight days [15]. The Australian Institute of Health and Welfare reported that the ALOS for MBD in Australian public hospitals (from 11 to 13 days) was longer than for private hospitals (from 5 to 6 days) [7,8,9,10,11,12]. The ALOS for MBD in public hospitals was about three times those for overall diagnoses (between 3 and 4 days) [7,8,9,10,11,12]. The current study showed that the mean and median ALOS for postpartum MBD was eight and five days respectively in NSW. It was shorter than that for overall MBD in Australian public hospitals (from 11 to 13 days) and this may be accounted for by the mothers’ desire for early discharge in order to be reunited with their infant. This would fit with the current lack of public mother—baby beds in NSW, whereby mothers are admitted to general psychiatric units without their infants. Twenty-six percent of mothers were admitted to hospital for postpartum MBD (principal diagnoses) more than once. The entire length of hospital stay in the first year postpartum for MBD (principal diagnoses) was eleven days for mean and six days for median. 

The hospital admissions with MBD as non-principal diagnoses should also be considered when assessing admission and length of hospital stay for MBD though there has been little discussion about this in the literature [22,29]. There are a number of reasons for this. Firstly, stay or other diagnoses did not suggest the MBD was less severe than principal diagnoses. Principal diagnoses, such as obstructed labour, postpartum haemorrhage and fatigue, were urgent problems and more likely to be diagnosed as principal diagnoses. Secondly, MBD extended the length of hospital stay of non-MBD principal diagnoses. For the principal diagnoses comorbid with MBD, their ALOS was significantly longer (six days) than general diagnoses (four days) in NSW [7]. Thirdly, this study showed that more mothers were admitted to hospital with MBD as stay or other diagnoses than principal diagnoses. The number was too large to be ignored. 

The mean of ALOS for postpartum MBD declined significantly from eleven to seven days between 2000 and 2005 (see Table 3). This was consistent with the slight fall in ALOS for public (3.9 days in 2004–2005 to 3.7 days in 2008–2009) and private hospitals (2.6 days in 2004–2005 to 2.4 days in 2008–2009) in Australia [30]. The changes in ALOS reflected a change in service delivery arrangements, such as a shift from psychiatric hospitals to general hospitals. The National Mental Health Strategy commitment in Australia (1993–2008) was to reduce the size of stand-alone psychiatric hospitals and develop more inpatient services within general hospitals [31,32]. This study showed that the mean ALOS declined significantly between 2000 and 2005 but median ALOS did not change significantly (see Table 3). It reflected that the relatively longer hospital stays such as those in psychiatric hospitals has been decreased. On the other hand, the hospital admissions for unipolar depressions, adjustment disorders and anxiety disorders significantly increased in the period (see Figure 2). This increase may be associated with the Beyondblue National Postnatal Depression Program (NPDP) (2001–2005) which increased awareness of perinatal depressions in health professionals and perinatal women through screening and education [33]. Given the ongoing investment in early intervention programs for perinatal mental health in Australia [34,35], and in New South Wales specifically, it will be important to continue to monitor features of postnatal hospital admissions for depression and related disorders. The NPDP is to be implemented in the following years in Australia [36]. The hospital admissions for depression need to be monitored over the coming years.

A large number of patient days for postpartum MBD were attributed to unipolar depression, adjustment disorder, acute psychotic episode and schizophrenia, schizophrenia-like disorder. There were large numbers of admissions for unipolar depression and adjustment disorder (65%), while schizophrenia, schizophrenia-like disorder and acute psychotic episode had long hospital stays (18.73–22.40 days). This is in keeping with the differential diagnostic burden *i.e.* more severe diagnoses (e.g. schizophrenia) will require longer admissions. The four diagnostic categories (unipolar depressions, adjustment disorders, acute psychotic episodes, and schizophrenia or schizophrenia-like disorder) accounted for almost 80% of patient days for MBD.

MDC and APDC are high quality data in completeness and accuracy. MDC cover all births and APDC cover all hospital admissions in NSW [37]. The datasets show a high level of concordance in the coding of delivery outcome (95.6%) and discharge status (99.1%) [38]. 

## 5. Limitations

There were a couple of limitations in the study. Firstly, the variable of hospital type was not available in this data so the impact of hospital type on ALOS could not be compared. Secondly, the data were limited to mothers who were admitted to hospital with MBD diagnoses and the ALOS for MBD in community service cannot be described.

## 6. Conclusions

There were 5,129 patient days of hospital stay in the first year after birth for principal diagnoses of postpartum MBD in new mothers in NSW between 2000 and 2005. MBD, especially unipolar depressions, adjustment disorders, acute psychotic episodes, and schizophrenia, schizophrenia-like disorders during the first year after birth, placed a significant burden on hospital services given the large number of admissions or long hospital stays.

## List of Abbreviations

ALOS: Average Length of Stay
APDC: Admitted Patients Data Collection
CHeReL: Centre for Health Record Linkage
CI: Confidence Interval
MBD: Mental and Behavioural Disorders
MDC: Midwives Data Collection
NHMRC: The National Health and Medical Research Council
NSW: New South Wales
US: United States
